# Association between obesity and fracture risk in Chinese women above 50 years of age: a prospective cohort study

**DOI:** 10.1186/s12889-023-17494-7

**Published:** 2024-01-02

**Authors:** Hui Li, Qunying Xu, Yunli Ye, Bei Chang, Rui Wang, Guangwen Li

**Affiliations:** 1 School of Public Health, Southwest Medical University, No. 1 Section 1, Xianglin Road, Longmatan District, Luzhou City, Sichuan Province 646000 China; 2 Department of Oral Implantology, The Affiliated Stomatological Hospital of Southwest Medical University, Oral & Maxillofacial Reconstruction and Regeneration of Luzhou Key Laboratory, No. 10, Section 2, Yunfeng Road, Kuanchang Street, Jiangyang District, Luzhou City, 646000 Sichuan Province China; 3 Institute of Stomatology, Southwest Medical University, Luzhou, 646000 China; 4 State Key Laboratory of Military Stomatology & National Clinical Research Center for Oral Diseases & Shaanxi Key Laboratory of Oral Diseases, School of Stomatology, The Fourth Military Medical University, Xi’an, 710032 China; 5 Chinese People’s Liberation Army Rocket Force Characteristic Medical Center, Beijing, 100000 China; 6 Tangdu Hospital, Air Force Military Medical University, Xi’an, 710032 China

**Keywords:** Body mass index, Fracture, Obesity, Waist circumference, Waist-to-height ratio

## Abstract

**Background:**

Fractures present serious health challenges for older adults, including premature mortality and reduced quality of life. Obesity has become significantly prevalent in China. However, the association between obesity and fractures remains unclear. This study aimed to assess the association between obesity and fractures among Chinese women above 50 years of age.

**Methods:**

A prospective cohort study was designed based on the China Health and Nutrition Survey, using data from 1997 to 2015. The average follow-up duration was seven years. Trained investigators measured body mass index (BMI) and waist circumference (WC) at baseline. Obesity was defined according to World Health Organization recommendations. Waist-to-height ratio (W-HtR) was calculated, with 0.5 as the cutoff value. Onset of fractures, self-reported by the participants during the follow-up period, was the primary outcome. Cox hazard regression models were used to assess the association between BMI, WC, W-HtR and subsequent risk of fracture. A sensitivity analysis was conducted by multiple imputation of missing data on the variables at baseline.

**Results:**

A total of 2,641 women aged ≥ 50 years were involved in the study. In all the models, no significant association existed between BMI and fracture risk. However, women with WC ≥ 88 cm had significantly higher risk of fracture than those with WC < 80 cm according to both the unadjusted (HR = 1.744, 95% CI: 1.173–2.591) and adjusted models (HR = 1.796, 95% CI: 1.196–2.695). In addition, W-HtR and fracture risk were positively associated according to both the unadjusted (HR = 1.798, 95% CI: 1.230–2.627) and adjusted models (HR = 1.772, 95% CI: 1.209–2.599). Results of the sensitivity analysis were consistent with those of the above analyses.

**Conclusions:**

Abdominal obesity increased the risk of all-cause fractures in Chinese women ≥ 50 years old. Intervention strategies and measures to prevent or address abdominal obesity would be helpful to decrease the fracture incidence.

## Introduction

Fractures are a health problem associated with premature mortality and reduced quality of life in older adults [1]. From 1990 to 2019, the absolute number of fractures worldwide increased by 33.4%, with older people involved in the majority of events [2]. Older women have a higher risk of fractures than do older men. In the China National Fracture Study, a nationally representative study including more than half a million people, women aged > 55 years had a significantly higher incidence of fracture than did women of other age groups and males of the same age group [3].

Multiple factors affect the risk of fractures. However, the association between obesity and fractures remains unclear. Several cohort studies have evaluated the relationship between obesity and fractures in older women in different regions worldwide. Even for the same fracture type, the results were conflicting. For example, for hip fractures, multiple studies have observed the protective role of obesity [4–7], whereas others have reported the risk effect of obesity based on a linear or U-shaped relationship [8–10]. The inconsistency in the results may be related to the heterogeneity of the population in terms of race, levels of obesity, and status of exposure to other risk factors for fracture (such as age, body composition, and lifestyle) [11].

The biological mechanisms underlying the impact of obesity on fractures are complex, and include biological and mechanical factors [12]. Excess fat mass plays an important role in bone loss by increasing the release of inflammatory cytokines, which are more pronounced in central obesity [13]. In addition, differences in the distribution of adipose tissue in the body may result in different mechanical mechanisms [13]; therefore, the impact of general and central obesity on fracture risk may differ [14].

In recent decades, the prevalence of obesity in China has increased significantly because of the acceleration of industrialisation and lifestyle transformation. According to the Report on Nutrition and Chronic Diseases in China (2020), more than 50% of adults assessed between 2015 and 2019 were overweight or obese [15]. In 2019, China had the second-highest disability-adjusted life years score owing to fractures related to low bone mass among 204 countries and territories [16]. Therefore, it is necessary to evaluate the relationship between obesity and fractures in China, where the population is ageing rapidly [17]. However, there is limited prospective research in China. In this study, we aimed to provide such relevant evidence from China. Based on a nationwide sample, a prospective study design was adopted to evaluate the association between general and abdominal obesity and fracture risk in Chinese women ≥ 50 years old.

## Methods

### Study population

The data for this study were obtained from the China Health and Nutrition Survey (CHNS), a longitudinal survey initiated in 1989 in China to observe how the social and economic transformation of Chinese society affects the health and nutritional status of the Chinese population [18]. Nine additional waves followed in 1991, 1993, 1997, 2000, 2004, 2006, 2009, 2011, and 2015. More than 30,000 participants from more than 7,200 households, comprising 15 provinces and autonomous cities or districts, participated in the study, were selected using multistage random cluster sampling [19]. The CHNS was approved by the Institutional Review Boards at the University of North Carolina at Chapel Hill and the National Institution for Nutrition and Food Safety, China Center for Disease Control and Prevention. All participants provided signed informed consent [18].

### Selection of participants

**Fig. 1:**
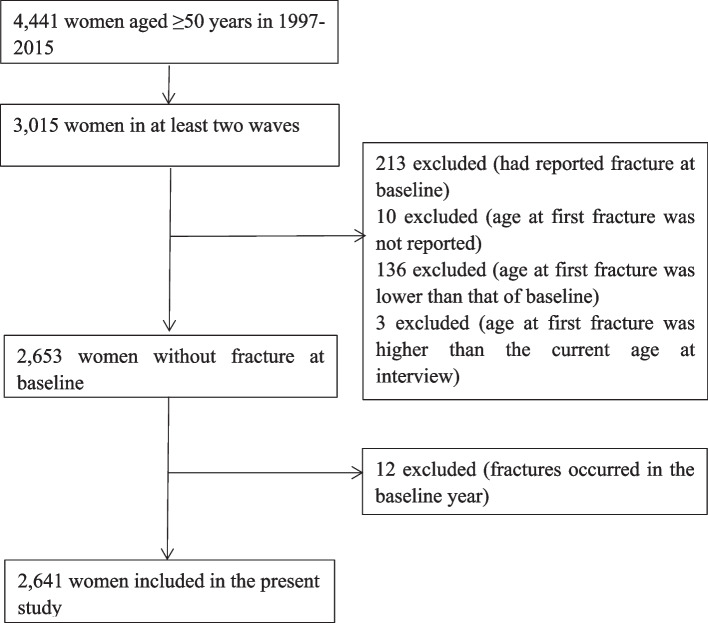
Selection procedure of study participants

In this study, data from 1997 to 2015 were used because fracture data were only available from 1997. A total of 2,641 women were included in this study. The selection procedure for the study participants is shown in Fig. 1.

Participants were included according to the following criteria: (1) women aged ≥ 50 years; (2) had participated in at least two waves; (3) had never undergone a fracture at baseline; (4) had reported their age at first fracture; and (5) were fracture-free at baseline year.

### Measure of exposure

Height, weight, and waist circumference (WC) of the participants were determined. The height of the participants was measured without footwear, using a portable SECA stadiometer (SECA, Hamburg, Germany) accurate to 0.1 cm [20]. The weight of the participants (who wore light clothing and no footwear), was measured using a calibrated beam scale (SECA882) before 2015 and a body composition tester (TANITA BC601) in 2015 with a measurement accuracy of 0.1 kg [20]. WC was measured to the nearest 0.1 cm above the navel with the participant breathing naturally and standing upright, using a Seca201 non-elastic tape [21]. Body mass index (BMI) was calculated as weight in kg divided by height in metres squared (m^2^). To enhance comparability of our findings with those from other countries, the World Health Organization’s recommendations for overweight and obesity were used. The BMI was classified into underweight (< 18.5 kg/m^2^), normal (18.5 kg/m^2^–24.9 kg/m^2^), overweight (25.0 kg/m^2^–29.9 kg/m^2^), and obese (≥ 30 kg/m^2^) [22]. WC was categorised as normal (< 80 cm), pre-obese (80–87.9 cm), or abdominal obesity (≥ 88 cm) [23]. In this study, waist-to-height ratio (W-HtR) was calculated as the ratio of WC to height. The cutoff value of W-HtR was defined as 0.5 [24].

### Measure of outcome

The onset of fractures was the primary outcome of this study. During each wave, the investigators asked the participants whether they had undergone fractures, and if so, the total number of fractures, and their age at the time of the first fracture. Participants who did not report having undergone a fracture during any wave were considered to have undergone no fractures. Among women who reported fractures, those who could not recall their age at the time of the first fracture were excluded. For any wave, participants reporting the first fracture at an age lower than the baseline age or higher than the current age at the time of the interview were also excluded. Among the remaining participants who had undergone a fracture, the first recalled age of the first fracture was used as the time of occurrence of the outcome to reduce information bias. The follow-up period considered was the number of years from the participant’s first interview to the endpoint or last interview.

### Definition of covariates

The baseline information of the following factors, which were measured using a structural questionnaire, was adopted as covariates: demographic factors (age, nationality, marital status, education level, income, place of residence, and wave); lifestyle behaviours (smoking, alcohol consumption, and physical activity); personal disease history (hypertension and/or diabetes); and dietary intake (energy, protein, fat, and carbohydrates). Family income per capita was inflated to values prevalent in 2015, and was then adopted as an indicator of income. Place of residence was recorded as rural or urban. Smoking status was recorded with the following question: “Have you ever smoked cigarettes?” (yes or no). Alcohol consumption was recorded via the query: “Did you drink beer or any other alcoholic beverage last year?” (yes or no). Physical activity was measured based on occupational, home, transportation, and leisure activities. The weekly consumption of the metabolic equivalent of task (MET) hours was used to measure the physical activity of the participants, calculated by multiplying the MET per hour and the duration (hours) per week of the activities. The MET per hour for each physical activity type was obtained from the Compendium of Physical Activities [25]. To obtain the daily dietary intake of energy (kcal), protein (g), fat (g), and carbohydrates (g), the dietary records method of three consecutive days was used [26]. In the present study, menopause status could not be included since CHNS did not collect information regarding menopause in adults from 1997.

### Statistical analysis

Skewed continuous variables were described as medians (interquartile ranges). Quantitative variables were described as frequencies and proportions. The distributions of covariables among the groups of BMI, WC, and W-HtR were compared using the rank sum test (for skewed distribution quantitative data) or the chi-squared test (for qualitative data). The log-rank test was used to compare fracture incidences among the exposure groups for each covariable. Factors associated with both anthropometric indexes (BMI, WC, and W-HtR) and fracture incidence were considered potential confounders, with the screening criteria set at *P* ≤ 0.1. The Cox hazard regression model was used to assess the association between obesity and subsequent risk of fracture. Hazard ratio (HR) was calculated using unadjusted and adjusted models that included the potential confounders mentioned above.

Owing to the missing baseline values of some variables, a sensitivity analysis was conducted. We imputed the missing data of the variables in Table 1 under the missing-at-random assumption using multiple imputation with a regression switching approach (chained equations with *m* = 10). The imputation procedure was performed using a linear regression method for continuous variables, and an ordinal or binary logistic regression model for categorical variables. Partial regression coefficients with 95% CI were combined with those in different imputed datasets using Rubin’s rules [27].

**Table 1:** Baseline characteristics of the participants

Variables	*n*(%) /Median (IQR)^a^
Age at baseline (year)	60 (54, 66)
Geographic region	
Rural	1,166 (44.2)
Urban	1,475 (55.9)
Survey year	
1997	1,238 (46.9)
2000	236 (8.9)
2004	206 (7.8)
2006	109 (4.1)
2009	204 (7.7)
2011	648 (24.5)
Nationality^b^	
Han	2,396 (91.1)
Others	235 (8.9)
Education level^b^	
Junior high school and below	2,272 (86.1)
Senior high school or above	368 (13.9)
Marital status^b^	
Unmarried	46 (1.7)
Married	2,004 (76.5)
Divorced/separated/widowed	570(21.8)
Annual household income per capita (yuan/year)^b^	5,868 (2,718, 12,694)
Smoking	
no	2,460 (93.1)
yes	181 (6.9)
Alcohol consumption^b^	
no	2,353 (89.4)
yes	280 (10.6)
Physical activity (METs/wk)	57.2 (24.4, 136.6)
Hypertension^b^	
no	2,159 (82.0)
yes	474 (18.0)
Diabetes^b^	
no	2,511(95.2)
yes	126 (4.8)
Energy intake (kcal/d)^b^	1,811 (1,464, 2,224)
Fat intake (g/d)^b^	57.6 (38.6, 81.3)
Protein intake (g/d)^b^	56.8 (45.1, 71.1)
Carbohydrate intake (g/d)^b^	254.7 (188.0, 328.7)
BMI (kg/m^2^)^b^	23.4 (20.9, 26.1)
WC (cm)^b^	81.0 (74.0, 89.0)
W-HtR^b^	0.53 (0.48, 0.58)

*Abbreviation*: *MET* Metabolic equivalent of task

^a^Median (IQR): age at baseline, annual household income per capita, physical activity, daily intake of dietary energy, daily fat intake, daily protein intake, daily carbohydrate intake, body mass index (BMI), waist circumference (WC) and waist-height ratio (W-HtR)

^b^There were 10 (0.4%) cases that were missing information for nationality, 21 (0.8%) for marriage, 1 (0.01%) for education level, 45 (1.7%) for annual household income per capita, 8 (0.3%) for alcohol drinking, 8 (0.3%) for hypertension, 4 (0.2%) for diabetes, 73 (2.8%) for energy intake, 73 (2.8%) for fat intake, 73 (2.8%) for protein intake, 73 (2.8%) for carbohydrate intake, 173 (6.6%) for BMI, 195 (7.4%) for WC, and 200 (7.6%) for W-HtR

Statistical analyses were performed using STATA 12.0, using a two-tailed test with significance set at 0.05.

## Results

### Distribution of basic characteristics among participants

The basic characteristics of the participants are shown in Table 1. The median age of the participants was 60 (54, 66) years. The median BMI, WC, and W-HtR at baseline were 23.4 (20.9, 26.1) kg/m^2^, 81.0 (74.0, 89.0) cm, and 0.53 (0.48, 0.58), respectively (Table 1).

Analysis of BMI of the participants showed that compared with underweight women, normal weight, overweight, or obese women were more likely to live in urban areas, be of Han nationality, be educated up to senior high school level or above, be married, be non-smokers, and have a history of hypertension and diabetes (all *P* < 0.05). They were also younger, had a higher income, and had a higher dietary intake of fat and protein, but a lower dietary intake of carbohydrates (all *P* < 0.05) (Table 2).

**Table 2:** Distribution of demographic factors, lifestyle behaviours, personal disease history, and dietary intake across BMI, WC and W-HtR groups

Variables		BMI (kg/m2)			*P* value		WC (cm)		*P* value	WHtR		*P* value
	< 18.5	18.5–24.9	25–29.9	≧30		< 80	80–87.9	≧88		≧0.5	< 0.5	
Age(year), median(IQR)^a^	64 (57, 71)	60 (54, 67)	59 (54, 64)	59 (54, 64)	** < 0.001**	59 (54, 66)	59 (54, 65)	60 (56, 66)	0.076	59 (54, 66)	60 (55, 66)	**0.004**
Geographic region, n(%)^b^												
Urban	56 (30.4)	638 (43.8)	356 (51.2)	70 (52.6)	** < 0.001**	414 (39.1)	342 (50.0)	362 (51.6)	** < 0.001**	359 (41.9)	807 (45.2)	0.105
Rural	128 (69.6)	818 (56.2)	339 (48.8)	63 (47.4)		646 (60.9)	342 (50.0)	340 (48.4)		498 (58.1)	977 (54.8)	
Survey year, n(%)^b^												
1997	119 (64.7)	698 (47.9)	233 (33.5)	44 (33.1)	** < 0.001**	595 (56.1)	247 (36.1)	229 (32.6)	** < 0.001**	464 (54.1)	774 (43.4)	** < 0.001**
2000	18 (9.8)	129 (8.9)	68 (9.8)	18 (13.5)		83 (7.8)	71 (10.4)	79 (11.2)		71 (8.3)	165 (9.2)	
2004	15 (8.1)	107 (7.3)	66 (9.5)	9 (6.8)		79 (7.5)	59 (8.6)	60 (8.6)		62 (7.2)	144 (8.1)	
2006	4 (2.2)	59 (4.1)	32 (4.6)	6 (4.5)		40 (3.8)	22 (3.2)	40 (5.7)		30 (3.5)	79 (4.4)	
2009	12 (6.5)	130 (8.9)	51 (7.3)	6 (4.5)		66 (6.2)	70 (10.2)	64 (9.1)		64 (7.5)	140 (7.9)	
2011	16 (8.7)	333 (22.9)	245 (35.3)	50 (35.6)		197 (18.6)	215 (31.4)	230 (32.8)		166 (19.4)	482 (27.0)	
Nationality, n(%)^b^												
Han	152 (82.6)	1301 (89.8)	654 (94.4)	131 (98.5)	** < 0.001**	913 (86.5)	637 (93.5)	669 (95.4)	** < 0.001**	747 (83.4)	1649 (92.9)	** < 0.001**
Others	32 (17.4)	148 (10.2)	39 (5.6)	2 (1.5)		142 (13.5)	44 (6.5)	32 (4.6)		108 (12.6)	127 (7.1)	
Education level, n(%)^b^												
Junior high school and below	173 (94.0)	1243 (85.4)	571 (82.2)	120 (90.2)	** < 0.001**	939 (88.7)	548 (80.1)	600 (85.5)	** < 0.001**	733 (85.6)	1539 (86.3)	0.659
Senior high school or above	11 (6.0)	212 (14.6)	124 (17.8)	13 (9.8)		120 (11.3)	136 (19.9)	102 (14.5)		123 (14.4)	245 (13.7)	
Marital status, n(%)^b^												
Unmarried	2 (1.1)	30 (2.1)	10 (1.4)	1 (0.7)	** < 0.001**	20 (1.9)	15 (2.2)	8 (1.2)	**0.001**	18 (2.1)	28 (1.6)	0.145
Married	105 (58.0)	1112 (77.0)	569 (82.1)	110 (84.0)		773 (73.8)	553 (81.2)	553 (79.1)		628 (74.2)	1376 (77.6)	
Divorced/separated/widowed	74 (40.9)	302 (20.9)	114 (16.5)	20 (15.3)		254 (24.3)	113 (16.6)	138 (19.7)		200 (23.6)	370 (20.8)	
Annual household income per capita (yuan/year), median(IQR)^a^	3000 (1666, 6172)	5753 (2768, 12,467)	7514 (3917, 16,425)	7105 (4042, 14,393)	** < 0.001**	4635 (2347, 9569)	7143 (3377, 16,202)	7368 (3742, 15,652)	** < 0.001**	5107 (2512, 11,190)	6245 (2855, 13,463)	**0.002**
Smoking, n(%)^b^												
no	161 (87.5)	1,360 (93.4)	657 (94.5)	126 (94.7)	**0.007**	968 (91.3)	650 (95.0)	662 (94.3)	**0.004**	782 (91.2)	1678 (94.1)	**0.007**
yes	23 (12.5)	96 (6.6)	38 (5.5)	7 (5.3)		92 (8.7)	34 (5.0)	40 (5.7)		75 (8.8)	106 (5.9)	
Alcohol drinking, n(%)^b^												
no	158 (86.3)	1301 (89.5)	615 (88.9)	122 (91.7)	0.441	936 (88.5)	607 (88.9)	637 (90.9)	0.26	752 (88.1)	1601 (90.0)	0.131
yes	25 (13.7)	152 (10.5)	77 (11.1)	11 (8.3)		122 (11.5)	76 (11.1)	64 (9.1)		102 (11.9)	178 (10.0)	
Physical activity(METs/wk), median(IQR)^a^	47.2 (13.3, 163.4)	64.2 (26.1, 142.6)	56.3 (27.1, 118.7)	54.2 (27.6, 131.5)	0.226	65.1 (24.4, 163.5)	59.9 (27.0, 126.3)	54.2 (25.9, 116.8)	**0.01**	65.3 (26.1, 158.7)	54.5 (23.8, 128.3)	**0.001**
Hypertension, n(%)^b^												
no	177 (96.7)	1242 (85.6)	511 (73.6)	81 (60.9)	** < 0.001**	941 (89.3)	556 (81.4)	493 (70.2)	** < 0.001**	764 (89.8)	1395 (78.3)	** < 0.001**
yes	6 (3.3)	209 (14.4)	183 (26.4)	52 (39.1)		113 (10.7)	127 (18.6)	209 (29.8)		87 (10.2)	387 (21.7)	
Diabetes, n(%)^b^												
no	180 (98.4)	1400 (96.3)	643 (92.6)	125 (94.0)	** < 0.001**	1032 (97.6)	648 (94.7)	646 (92.2)	** < 0.001**	834 (97.7)	1677 (94.0)	** < 0.001**
yes	3 (1.6)	54 (3.7)	51 (7.4)	8 (6.0)		25 (2.4)	36 (5.3)	55 (7.8)		20 (2.3)	106 (6.0)	
Energy intake (kcal/d), median(IQR)^a^	1867 (1488, 2319)	1806 (1460, 2234)	1811 (1470, 2187)	1820 (1456, 2228)	0.654	1813 (1487, 2262)	1802 (1440, 2203)	1806 (1441, 2185)	0.254	1823 (1463, 2240)	1810 (1466, 2218)	0.524
Fat intake (g/d), median(IQR)^a^	51.3 (31.5, 78.7)	56.8 (38.3, 80.4)	60.9 (43.0, 82.9)	65.1 (38.9, 87.1)	**0.001**	56.6 (36.9, 79.5)	58.4 (41.7, 83.0)	59.4 (40.0, 82.3)	0.054	56.3 (36.4, 78.9)	58.0 (39.8, 82.6)	0.086
Protein intake (g/d), median(IQR)^a^	53.7 (44.4, 64.8)	56.4 (43.9, 70.5)	59.3 (48.2, 73.1)	58.6 (46.5, 74.2)	** < 0.001**	55.5 (43.9, 69.3)	57.8 (44.6, 73.0)	58.4 (47.3, 72.1)	**0.002**	56.1 (44.4, 70.5)	57.1 (45.3, 71.3)	0.154
Carbohydrate intake (g/d), median(IQR)^a^	285.3 (218.7, 358.9)	254.9 (188.6, 333.9)	242.9 (174.5, 309.8)	249.2 (179.1, 315.1)	** < 0.001**	263.7 (196.2, 345.2)	241.1 (181.7, 318.4)	243.5 (177.7, 312.8)	** < 0.001**	261.3 (192.6, 343.5)	250.7 (185.2, 322.4)	**0.015**

The bold front indicated the *P* value was less than or equal to 0.05

*Abbreviation*: *BMI* Body mass index, *WC *Waist circumference, *W-HtR *Weight to height ratio, *MET *Metabolic equivalent of task

^a^by Kruskal–Wallis test for BMI and WC, Mann–Whitney U test for W-HtR b: by chi-square test

Analysis of WC of the participants showed that compared with women of normal weight, pre-obese and obese women were more likely to live in cities, be of Han nationality, be educated up to junior high school or above, be married, be non-smokers, and have a history of hypertension and diabetes (all *P* < 0.05). They also had a higher income, a lower intensity of physical activity, a higher dietary intake of protein, but a lower dietary intake of carbohydrates (all *P* < 0.05) (Table 2).

Compared with women whose W-HtR ≤ 0.5, women with W-HtR > 0.5 were more likely to be younger, be of Han nationality, be non-smokers, and have a history of hypertension and diabetes (all *P* < 0.05). They also had higher income, a lower intensity of physical activity, and a lower dietary intake of carbohydrates (all *P* < 0.05) (Table 2).

In addition, the distribution of waves at baseline differed significantly among the BMI, WC, and W-HtR groups (all *P* < 0.05) (Table 2).

### Association between basic characteristics and fracture risk

The median (interquartile range) follow-up period was 7.0 (4.0, 14.0) years with 22,977 person-years. A total of 149 fractures were reported during follow-up. Women living in urban areas, educated up to junior high school education or below, and with a high dietary intake of fat had a significantly higher risk of fractures (all *P* < 0.05). Women aged ≥ 60 years or with a higher dietary energy intake had a marginal higher risk of fracture (all *P* < 0.1) (Table 3).

**Table 3:** Distribution of fracture incidence among women by characteristics

Variables	Incidence (no. of fractures/ 1000 person-years)	χ^2^ value	*P* value
Age(year)			
< 60	5.5	3.76	0.052
≧60	7.6		
Geographic region			
Urban	8.6	9.70	**0.002**
Rural	5.2		
Survey year			
1997	7.1	11.18	**0.048**
2000	9.0		
2004	5.4		
2006	2.6		
2009	4.3		
2011	3.1		
Nationality			
Han	6.7	1.68	0.195
Others	4.6		
Education level			
Junior high school and below	6.8	3.07	**0.080**
Senior high school or above	3.6		
Marital status			
Unmarried	11.2	1.73	0.421
Married	6.1		
Divorced/separated/widowed	6.6		
Annual household income per capita (yuan/year)			
Q1	6.6	0.87	0.834
Q2	6.7		
Q3	6.6		
Q4	5.2		
Smoking			
No	6.7	1.28	0.259
Yes	4.5		
Alcohol consumption			
no	6.7	0.85	0.357
yes	5.1		
Physical activity, METs/wk			
Q1	6.6	1.77	0.620
Q2	5.8		
Q3	7.5		
Q4	6.1		
Hypertension			
no	6.3	0.79	0.373
yes	7.6		
Diabetes			
no	6.5	0.01	0.919
yes	6.2		
Energy intake (kcal/d)			
Q1	4.6	6.91	0.075
Q2	6.3		
Q3	8.7		
Q4	6.0		
Fat intake (g/d)			
Q1	6.1	9.33	**0.025**
Q2	5.5		
Q3	5.3		
Q4	9.4		
Protein intake (g/d)			
Q1	5.1	2.70	0.440
Q2	7.5		
Q3	7.1		
Q4	6.3		
Carbohydrate intake (g/d)			
Q1	7.3	2.56	0.464
Q2	5.6		
Q3	7.6		
Q4	6.0		

The bold front indicated the* P* value was less than or equal to 0.05

*Abbreviation*: *MET* Metabolic equivalent of task

### Association between BMI and fracture risk

Fracture incidence among participants with BMI < 18.5 kg/m^2^, 18.5–24.9 kg/m^2^, 25–29.9 kg/m^2^, and ≥ 30 kg/m^2^ was 5.7, 5.8, 7.6, and 9.2 per 1000 person-years, respectively. First, we assessed the association between BMI and fracture risk without including missing data. There was no association between BMI and fracture risk in either the unadjusted (model 1) or adjusted (model 2) models. Similarly, after imputing the missing baseline data for the variables shown in Table 1, no significant association was observed between BMI and fracture risk in either the unadjusted (model 3) or adjusted (model 4) models (Table 4).

**Table 4:** Association between overweight, obesity, and fracture risk among Chinese women above 50 years of age

Variables	Incidence (no. of fractures/ 1000 person-years)	Model 1^a^	Model 2^b^	Model 3^c^	Model 4^d^
		HR (95% CI)	HR (95% CI)	HR (95% CI)	HR (95% CI)
BMI (kg/m^2^)					
< 18.5	5.7	0.988 (0.511, 1.911)	0.903 (0.465, 1.753)	0.987 (0.514, 1.893)	0.890 (0.460, 1.723)
18.5–24.9	5.8	1.000	1.000	1.000	1.000
25–29.9	7.6	1.325 (0.905, 1.940)	1.375 (0.933, 2.026)	1.316 (0.895, 1.934)	1.338 (0.906, 1.976)
≧30	9.2	1.619 (0.810, 3.236)	1.503 (0.748, 3.018)	1.566 (0.785, 3.124)	1.461 (0.727, 2.934)
WC (cm)					
< 80	5.1	1.000	1.000	1.000	1.000
80–87.9	6.3	1.238 (0.803, 1.910)	1.235 (0.793, 1.923)	1.197 (0.784, 1.829)	1.221 (0.792, 1.882)
≧88	8.8	**1.744 (1.173, 2.591)**	**1.796 (1.196, 2.695)**	**1.680 (1.137, 2.482)**	**1.704 (1.143, 2.541)**
W-HtR					
≤ 0.5	4.3	1.000	1.000	1.000	1.000
> 0.5	7.7	**1.798 (1.230, 2.627)**	**1.772 (1.209, 2.599)**	**1.701 (1.160, 2.495)**	**1.693 (1.149, 2.494)**

The bold front indicated the 95% *CI* excluded 1

*Abbreviation*: *BMI* Body mass index, *WC* Waist circumference, *W-HtR* Waist-to-height ratio

^a^Unadjusted model without imputation

^b^Adjusted models without imputation: adjusted for age, wave, geographic region, education level and dietary fat intake on the association between BMI, WC and fracture risk; adjusted for age, wave and dietary fat intake on the association between W-HtR and fracture risk

^c^Unadjusted model with imputation

^d^Adjusted models with imputation: adjusted for age, wave, geographic region, education level and dietary fat intake on the association between BMI, WC and fracture risk; adjusted for age, wave, geographic region and dietary fat intake on the association between W-HtR and fracture risk

### Association between WC and fracture risk

Fracture incidence among participants with WC < 80 cm, 80–87.9 cm, and ≥ 88 cm was 5.1, 6.3, and 8.8 per 1000 person-years, respectively. Without imputation of missing data at baseline, and considering WC < 80 cm as a reference, a significant increase in fracture risk was observed for the group with WC ≥ 88 cm in both the unadjusted (model 1: HR = 1.744, 95% CI: 1.173–2.591) and adjusted models (model 2: HR = 1.796, 95% CI:1.196–2.695) after adjusting for age, wave, geographic region, education level, and dietary fat intake. After imputation of missing data at baseline, women with WC ≥ 88 cm had a significantly higher risk of fracture than did those with WC < 80 cm in both the unadjusted (model 3: HR = 1.680, 95% CI: 1.137–2.482) and adjusted models (model 4: HR = 1.704, 95% CI: 1.143–2.541) involving age, wave, geographic region, education level, and dietary fat intake (Table 4).

### Association between W-HtR and fracture risk

Fracture incidence among participants with W-HtR ≤ 0.5 was 4.3/1000 person-years, while those for women with W-HtR > 0.5 was 7.7/1000 person-years. Without imputation of missing data at baseline, the fracture risk was significantly higher for the W-HtR > 0.5 group than for the W-HtR ≤ 0.5 group, no matter the unadjusted (model 1: HR = 1.798, 95% CI: 1.230–2.627) or adjusted model (model 2: HR = 1.772, 95% CI: 1.209–2.599) after adjusting for age, wave, and dietary fat intake. After imputation of missing data at baseline, women with W-HtR > 0.5 had a significantly higher risk of fracture than did those with W-HtR ≤ 0.5 in both the unadjusted (model 3: HR = 1.701, 95% CI: 1.160–2.495) and adjusted models (model 4: HR = 1.693, 95% CI: 1.149–2.494) involving age, wave, and dietary fat intake (Table 4).

## Discussion

In this study, we did not observe a significant association between BMI and all-cause fracture risk, whereas positive associations between WC, W-HtR, and fracture risk were observed among Chinese women above 50 years of age.

Obesity was originally thought to reduce the risk of fractures, owing to a higher bone mineral density (BMD) in obese individuals, and the protective role of soft tissue padding against falls [28]. However, this viewpoint has been challenged by several epidemiological studies, especially those in postmenopausal women, which have observed a positive association between obesity and fracture [29–31]. The mechanism underlying obesity-related fractures is also controversial. In addition to the higher BMD owing to mechanical loading [28], oestrogen synthesis mechanisms may help postmenopausal obese women maintain bone homeostasis. After menopause, oestrogen biosynthesis is catalysed by aromatase, mainly in the adipose tissue, which converts adrenal androgens into oestrogens [32]. This promotes osteoclast apoptosis, osteogenesis, and mesenchymal stem cell differentiation, while inhibiting osteoclastogenesis from preosteoblasts to osteoblasts [33].

However, studies have also revealed the negative effects of obesity on bones. First, obese individuals have a higher risk of falls [34]. Moreover, multiple epidemiological studies have found that obese individuals have lower vitamin D concentrations than do non-obese individuals [35]. The underlying mechanisms may include volumetric dilution, sequestration of vitamin D into adipose tissue, limited sunlight exposure, and decreased vitamin D synthesis in vivo [35]. In addition, the hypermetabolic status of bone marrow stromal cells, and an accelerated senescent bone marrow microenvironment (such as expanded bone marrow adipose tissue) in obese individuals, leads to increased bone fragility [36].

In our study, we did not observe an association between BMI and fracture risk in women ≥ 50 years old. In a meta-analysis of 12 cohort studies, of which 11 used BMI as the obesity level indicator, overweight and obesity was associated with an decreased risk of all-cause fractures in postmenopausal women (HR = 0.86, 95% CI: 0.77–0.97) [11]. However, a recent cohort study with 456,921 participants from the UK biobank revealed a U-shape relationship between BMI and fracture, with the lowest risk of fracture in overweight participants [14].

In the present study, we noted that the all-cause fracture incidence was similar for both underweight and normal weight women, which then increased with BMI. The lack of an association between BMI and fracture risk may be related to the limitations of the sample size. In our study, the median BMI of the participants was 23.4 kg/m^2^, and the proportion of obese participants was only 5.39%, with a highest BMI value of 37.5 kg/m^2^. Therefore, the association between BMI and fracture risk in Chinese women above 50 years of age may need to be evaluated in a larger sample.

However, it is worth noting that the BMI has certain limitations. It cannot distinguish individuals with excess body fat from those with high muscle mass. And it cannot reflect the characteristics of fat distribution either [24]. Therefore, when assessing the relationship between BMI and fracture risk, different body fat mass and fat distribution among study populations may lead to different observations among studies.

In the present study, apart from WC, W-HtR, which has a superiority over WC and BMI for detecting cardiometabolic risk factors in adults, was used to assess abdominal obesity [37]. The results showed that abdominal obesity significantly increased the risk of fracture in all models based on either WC or W-HtR. This finding is consistent with the results of several previous studies. A recent prospective study in Iran found that postmenopausal women with WC ≥ 95 cm had a significantly higher risk of incident-hospitalised fracture (HR = 2.43, 95% CI: 1.53–3.86) [38]. In Zhu’s study [14], a linear positive correlation between WC adjusted for BMI and fracture risk was observed in older women (HR = 1.02, 95% CI: 1.01–1.02, *P* = 1.72E-11), which was supported by leveraging genetic instrumental variables.

Abdominal obesity is characterised by excessive visceral fat and is often accompanied by metabolic disorders [39]. The crucial role of cytokines (such as TNF-ɑ, IL-6) produced in adipose tissue in increasing the risk of osteoporosis has been revealed [12]. TNF-ɑ could promote the production of osteoclasts, synergise with cytokine RANKL, facilitate RANK-RANKL binding [40], and up-regulate the expression of RANKL, which promotes resorptive activity of osteoclasts [41]. IL-6 promotes osteoclast production and bone resorption by stimulating mesenchymal progenitor differentiation into the osteoblastic lineage and mediating the stimulatory effects of TNF [42]. Compared with subcutaneous fat, visceral fat secretes cytokines more actively. This could explain the increase in the risk of fractures with increased WC in postmenopausal women.

The distribution of fat tissue changes with age, being marked by increasing visceral adipose tissue and decreasing subcutaneous adipose tissue [12]. In this study, the median WC of the participants was 81 cm, and the proportion of abdominal obesity was 28.7%, suggesting that abdominal obesity may be more common than general obesity in Chinese women above 50 years of age. According to the data from the China PEACE Million Persons Project, from 2014 to 2018, the proportion of women with WC ≥ 85 cm in the 55–64 and 65–75 years old groups was 42.5% and 46.3%, respectively [43]. Thus, the population attributable risk proportion of abdominal obesity on fracture risk may be relatively high in Chinese women ≥ 50 years of age, which requires the attention of health departments.

The strength of our study is that it was based on a nationwide sample combined with a prospective study design to assess the association between obesity and fracture in Chinese women above 50 years of age. To the best of our knowledge, this is the first nationwide prospective study to assess the association between obesity and fracture risk in China. However, this study has some limitations. First, fracture history and age at first fracture were mainly self-reported by the participants; thus, information bias may exist. Second, we did not collect information regarding the fracture site. The associations between obesity and fracture may differ for different sites. Last, the study did not collect information on bone density or on the reason behind the first fracture; therefore, it was difficult to analyse the mechanism underlying the relationship between obesity and fracture.

## Conclusions

Abdominal obesity increased all-cause fracture risk for Chinese women ≥ 50 years, which suggests the importance of abdominal obesity intervention in preventing fracture in middle aged and older Chinese women. Further studies with larger sample sizes are required to elucidate the relationship between general obesity and fracture.

## Data Availability

The data that support the findings of this article are available from the public, open access website (https://www.cpc.unc.edu/projects/china/data/datasets).

## Abbreviations

BMD: Bone mineral density
BMI: Body mass index
CHNS: China Health and Nutrition Survey
HR: Hazard ratio
MET: Metabolic equivalent of task
WC: Waist circumference
W-HtR: Waist-to-height ratio
